# Hypersensitivity Pneumonitis-Like Drug-Induced Interstitial Lung Disease Caused by Pegylated Liposomal Doxorubicin: A Case Report

**DOI:** 10.7759/cureus.96827

**Published:** 2025-11-14

**Authors:** Tomoyuki Araya, Toshiyuki Kita, Takayuki Higashi, Ryo Hara, Hazuki Takato

**Affiliations:** 1 Respiratory Medicine, NHO Kanazawa Medical Center, Kanazawa, JPN

**Keywords:** corticosteroid therapy, drug-induced interstitial lung disease, granuloma, hypersensitivity pneumonitis, pegylated liposomal doxorubicin

## Abstract

Drug-induced interstitial lung disease (DI-ILD) encompasses a wide spectrum of imaging and histopathologic patterns, sometimes resembling hypersensitivity pneumonitis (HP). Pegylated liposomal doxorubicin (PLD, Doxil®) is a nanoparticulate formulation of doxorubicin designed to reduce systemic toxicity, and pulmonary toxicity is rarely documented. We report a 62-year-old woman with recurrent ovarian carcinoma who developed fever and erythema after the third cycle of PLD, followed by diffuse pulmonary infiltrates. She had no history of allergy, lung disease, or environmental antigen exposure. On admission, chest radiography revealed bilateral fine granular opacities, and high-resolution computed tomography demonstrated diffuse centrilobular ground-glass nodules predominantly in the upper lobes. Bronchoalveolar lavage showed marked lymphocytosis with elevated total cell counts, and transbronchial lung biopsy revealed noncaseating epithelioid granulomas with lymphocytic infiltration, consistent with an HP-like pattern. Microbiologic and autoimmune evaluations were negative. Based on the temporal association with PLD administration, compatible imaging and histopathologic findings, and exclusion of other etiologies, a diagnosis of HP-like DI-ILD induced by PLD was made. Prednisolone 50 mg daily led to rapid defervescence and remarkable radiologic improvement within two weeks, followed by complete remission after tapering and no recurrence during 4.5 months of follow-up until death from progressive ovarian cancer. This case highlights that PLD, though considered relatively safe, can induce HP-like DI-ILD, and prompt recognition with appropriate corticosteroid therapy may achieve favorable outcomes. Clinicians should remain vigilant for this rare but potentially severe pulmonary toxicity.

## Introduction

Drug-induced interstitial lung disease (DI-ILD) presents with a broad spectrum of radiologic and histopathologic manifestations caused by various cytotoxic and targeted anticancer agents [1]. Clinically, DI-ILD often presents with non-specific symptoms, such as fever, dry cough, and dyspnea, whereas auscultatory findings and sputum production are usually absent. Among these, hypersensitivity pneumonitis (HP)-like forms represent a particularly uncommon subtype, characterized by lymphocytic alveolitis and noncaseating granulomatous inflammation. Although HP-like reactions have been described with several agents, such as methotrexate, paclitaxel, docetaxel, pemetrexed, everolimus, and immune checkpoint inhibitors [2-7], they remain exceedingly rare in clinical practice. One of the major diagnostic challenges is differentiating drug-induced HP-like disease from infectious pneumonias, true HP, idiopathic interstitial pneumonias, or connective tissue disease-associated ILD. Moreover, histopathologic confirmation through bronchoalveolar lavage or lung biopsy is infrequently achieved, and establishing causality requires careful assessment of the clinical course, radiologic features, and exclusion of other etiologies.

Pegylated liposomal doxorubicin (PLD, Doxil®) is a long-circulating liposomal formulation of doxorubicin designed to reduce systemic toxicity, particularly cardiotoxicity and alopecia, by altering its pharmacokinetic profile. According to the latest Japanese package insert (Fuji Pharma Co., Ltd., February 2025 revision), interstitial lung disease and pneumonitis occur in approximately 1.4% of treated patients, indicating that pulmonary toxicity is rare [8]. To date, no HP-like form of DI-ILD associated with PLD has been reported worldwide. The present case is therefore of particular significance: it demonstrates, through a detailed clinical course with preceding cutaneous manifestations, radiologic findings on high-resolution computed tomography (HRCT), and histopathologic confirmation by bronchoalveolar lavage and transbronchial lung biopsy, that PLD can indeed cause HP-like DI-ILD. This case provides valuable evidence for clinicians when evaluating new-onset pulmonary infiltrates during PLD therapy.

## Case presentation

A 62-year-old woman with stage IIIB ovarian carcinoma had previously received three cycles of neoadjuvant chemotherapy with carboplatin and paclitaxel, followed by abdominal hysterectomy, bilateral salpingo-oophorectomy, and omentectomy. After surgery, she underwent six additional cycles of adjuvant chemotherapy with carboplatin and paclitaxel and subsequently received four months of maintenance therapy with niraparib. Disease progression was observed after four months of niraparib treatment. PLD was therefore initiated one month after the final dose of niraparib as third-line chemotherapy. She was a lifelong nonsmoker with no history of allergy or lung disease. There was no family history of pulmonary or allergic diseases. Environmental exposure included a 40-year-old wooden house and long-term use of feather bedding, but no humidifier, pet, or occupational dust exposure.

On day 20 of the third PLD cycle, she developed fever and erythematous axillary rashes. Erythema multiforme was diagnosed and treated with prednisolone 20 mg daily for seven days, with temporary improvement. Fever recurred on day 34, and chest radiography on day 39 revealed bilateral granular opacities, prompting admission.

**Figure 1:**
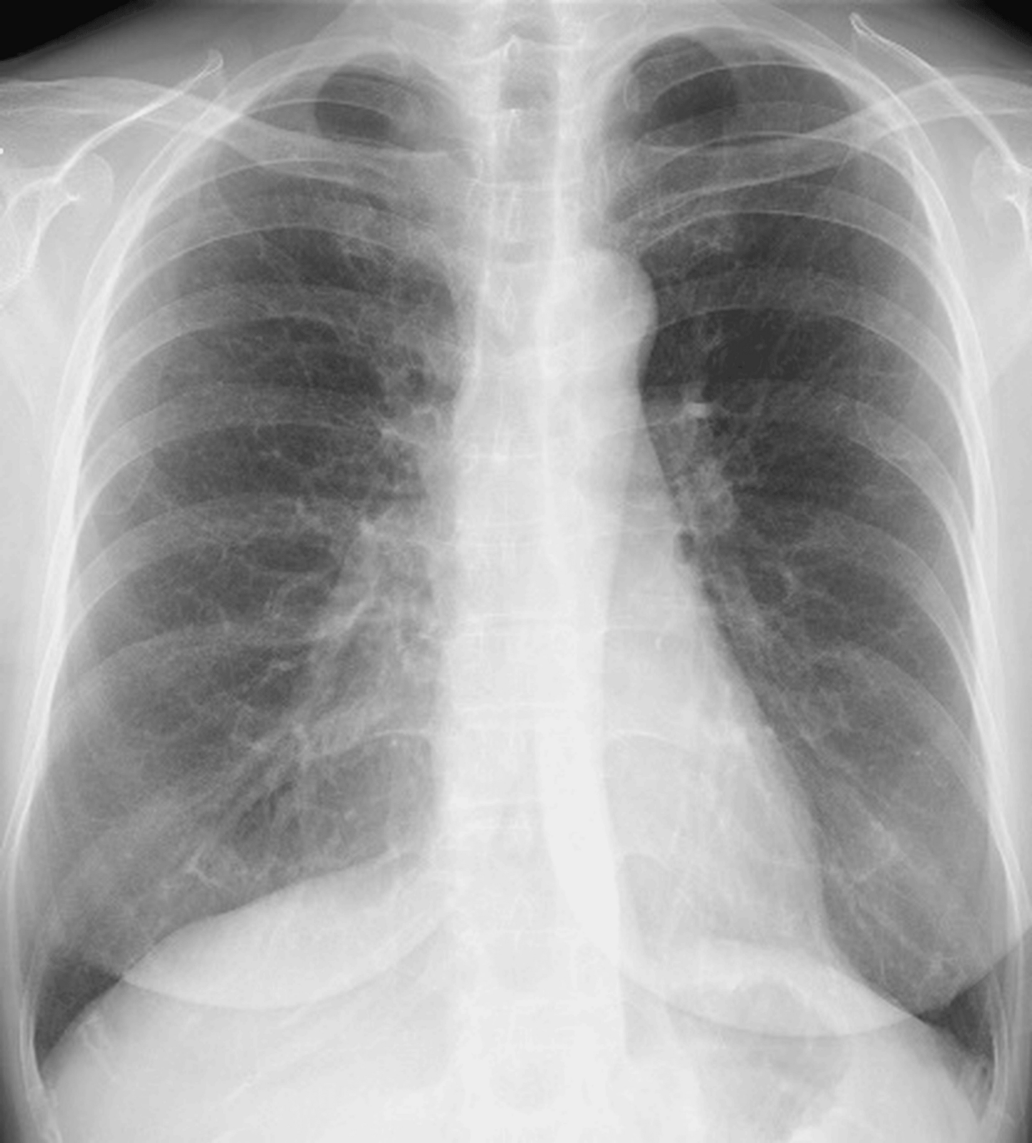
Chest radiograph on admission Chest radiograph showing bilateral fine granular opacities.

On admission, she was febrile (38.4 °C) but had no cough, sputum production, or dyspnea. Hemodynamics were stable, and oxygen saturation was normal (97% on room air). Examination revealed axillary erythema with pigmentation and epidermal peeling; lung sounds were normal. Chest radiography showed bilateral fine granular shadows (Figure 1).

**Figure 2:**
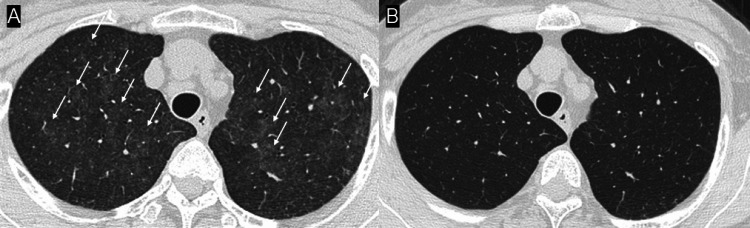
High-resolution computed tomography (HRCT) findings before and after corticosteroid therapy HRCT images showing diffuse centrilobular ground-glass nodules predominantly in the upper lobes (arrows), which markedly improved after corticosteroid therapy (prednisolone 50 mg/day). (A) On admission. (B) Two weeks after initiation of corticosteroid therapy.

HRCT demonstrated diffuse centrilobular ground-glass nodules, predominantly in the upper lobes (Figure 2A).

Laboratory results showed leukopenia (white blood cell count: 3,000/μL; neutrophils: 54%), elevated lactate dehydrogenase (249 U/L), and increased C-reactive protein (2.36 mg/dL). Serum levels of Krebs von den Lungen-6, surfactant protein-D, and surfactant protein-A were 454 U/mL, 55.0 ng/mL, and 130.1 ng/mL, respectively. Autoimmune and infectious evaluations - including antinuclear antibody, antineutrophil cytoplasmic antibody, anti-trichosporon asahii antibody, avian-specific immunoglobulin G, *Mycoplasma *antigen, polymerase chain reaction (PCR) for human immunodeficiency virus (HIV) antigen/antibody, and severe acute respiratory syndrome coronavirus 2 (SARS-CoV-2) - were all negative. Serum angiotensin-converting enzyme level was within the normal range (Table 1).

**Table 1:** Laboratory findings on admission Laboratory data revealed leukopenia, mild anemia, hypoalbuminemia, elevated C-reactive protein, and mild elevation of SP-A, while most infectious and autoimmune serologies were negative. Abbreviations: WBC, white blood cell count; Neu, neutrophil; Lym, lymphocyte; Mon, monocyte; Eos, eosinophil; Bas, basophil; RBC, red blood cell count; Hb, hemoglobin; Ht, hematocrit; Plt, platelet count; CRP, C-reactive protein; T-Bil, total bilirubin; TP, total protein; ALP, alkaline phosphatase; AST, aspartate aminotransferase; ALT, alanine aminotransferase; LDH, lactate dehydrogenase; Alb, albumin; Na, sodium; K, potassium; Cl, chloride; BUN, blood urea nitrogen; Cre, creatinine; eGFR, estimated glomerular filtration rate; UA, uric acid; BNP, brain natriuretic peptide; HbA1c, hemoglobin A1c; D-dimer, fibrin degradation product D-dimer; Ig, immunoglobulin; ACE, angiotensin-converting enzyme; KL-6, Krebs von den Lungen-6; SP-D, surfactant protein-D; SP-A, surfactant protein-A; β-D-glucan, beta-D-glucan; Ag, antigen; Ab, antibody; IGRA, interferon-γ releasing assay; ANA, antinuclear antibody; PR3, proteinase-3; MPO, myeloperoxidase; CMV, cytomegalovirus; HIV, human immunodeficiency virus; PCR, polymerase chain reaction; SARS-CoV-2, severe acute respiratory syndrome coronavirus 2; GPL, glycopeptidolipid

Parameter (unit)	Result	Normal range
WBC (/µL)	3.0×10³	4.5–9.0×10³
Neu (%)	54.0	38–74
Lym (%)	30.0	16.5–49.5
Mon (%)	14.0	5–10
Eos (%)	2.0	0–10
Bas (%)	0	0–2
RBC (×10⁴/µL)	319	382–500
Hb (g/dL)	10.3	11.7–14.6
Ht (%)	31.1	34.3–44.2
Plt (×10⁴/µL)	15.0	13–35
CRP (mg/dL)	2.36	0–0.4
T-Bil (mg/dL)	0.5	0.3–1.2
TP (g/dL)	6.0	6.7–8.3
ALP (U/L)	59	38–113
AST (U/L)	22	13–33
ALT (U/L)	14	6–27
LDH (U/L)	249	119–229
Alb (g/dL)	3.0	4.0–5.0
Na (mEq/L)	135	135–149
K (mEq/L)	4.0	3.5–4.9
Cl (mEq/L)	102	96–108
BUN (mg/dL)	14.6	8–22
Cre (mg/dL)	0.75	0.5–0.8
eGFR (mL/min/L)	60.1	60–100
UA (mg/dL)	2.7	2.3–7.0
BNP (pg/mL)	8.4	<18.4
HbA1c (%)	6.2	<6.4
D-dimer (µg/mL)	1.5	0–1
IgG (mg/dL)	792	870–1700
IgA (mg/dL)	252	110–410
IgM (mg/dL)	32	35–220
IgE (IU/mL)	62.0	<360.9
ACE (U/L)	14.7	7.7–29.4
KL-6 (U/mL)	454	<500
SP-D (ng/mL)	55.0	<109.9
SP-A (ng/mL)	130.1	<43.7
β-D-glucan (pg/mL)	3.523	<11.0
Aspergillus Ag	Negative	Negative
Cryptococcus Ag	Negative	Negative
IGRA	Negative	Negative
Anti-GPL core IgA	Negative	Negative
Mycoplasma PCR	Negative	Negative
SARS-CoV-2 PCR	Negative	Negative
HIV Ag/Ab	Negative	Negative
CMV antigenemia	Negative	Negative
ANA titer	<1:40	<1:40
PR3-ANCA (U/mL)	<1.0	<1.0
MPO-ANCA (U/mL)	<1.0	<1.0
Anti-trichosporon asahii Ab	Negative	Negative

Bronchoalveolar lavage from the right B5a segment revealed lymphocytosis (33-52%), elevated total cell counts (7.0-10.2 × 10⁵ cells/mL), and a cluster of differentiation (CD) 4/CD8 ratio of 1.6. Cultures and PCRs for bacteria, fungi, mycobacteria, and *Pneumocystis jirovecii *were negative. Cytology showed no pathogens or malignant cells.

**Figure 3:**
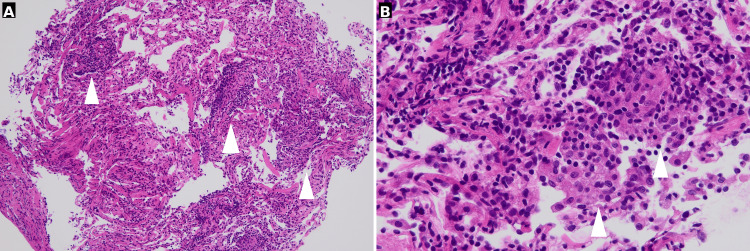
Histopathological findings of transbronchial lung biopsy specimens Transbronchial lung biopsy (hematoxylin and eosin staining; A: ×100; B: ×400) showing noncaseating epithelioid granulomas with lymphocytic infiltration. Arrowheads indicate representative granulomatous lesions composed of epithelioid histiocytes and lymphocytes.

Transbronchial biopsy from the right B2a segment demonstrated scattered noncaseating epithelioid granulomas with lymphocytic infiltration and no eosinophilia or vasculitis, consistent with HP (Figure 3).

Given the temporal association with PLD, compatible radiologic and histologic findings, and exclusion of other causes, a diagnosis of HP-like DI-ILD induced by PLD was made. The drug lymphocyte stimulation test (DLST) was negative.

Prednisolone 50 mg daily was initiated, leading to defervescence within one day and marked radiologic improvement within two weeks (Figure 2B). The dose was tapered weekly (30 → 20 → 10 mg). Corticosteroid therapy was discontinued after 3.5 months. No additional chemotherapy was administered after recovery, and no recurrence of pulmonary toxicity occurred during 4.5 months of follow-up until death from progressive ovarian cancer.

## Discussion

This report describes the first documented case of PLD-induced HP-like DI-ILD confirmed by bronchoalveolar lavage and transbronchial lung biopsy. Although HP-like DI-ILD has been reported with several chemotherapeutic and targeted agents, including methotrexate, docetaxel, and immune checkpoint inhibitors [2-7,9], it remains extremely uncommon in clinical practice. The pathogenesis is presumed to involve drug-triggered immune activation leading to alveolitis and granulomatous inflammation. However, diagnostic confirmation is often challenging because the presentation can mimic infectious pneumonia, idiopathic interstitial pneumonias, or connective tissue disease-related ILD, and histopathologic evaluation is not always feasible in compromised oncology patients.

In our case, the diagnosis was supported by several key features: (1) a clear temporal relationship with PLD administration, (2) subacute onset with fever and erythematous rash preceding pulmonary symptoms, (3) diffuse centrilobular ground-glass nodules on high-resolution computed tomography, (4) bronchoalveolar lavage showing lymphocytosis with elevated total cell counts, and (5) transbronchial biopsy demonstrating noncaseating granulomas with lymphocytic infiltration but no eosinophilia or vasculitis. These findings were highly consistent with an HP-like immune-mediated process rather than infectious or malignant etiologies. The absence of environmental antigen exposure and negative microbiologic and autoimmune studies further strengthened the causal inference.

To date, pulmonary toxicity from PLD has been described as extremely rare, with an incidence of approximately 1.4% according to the package insert [8], and only a few cases of non-specific interstitial pneumonitis have been reported [10]. The present case extends the spectrum of PLD-related pulmonary reactions by identifying an HP-like pattern, which had not been reported previously. Although a recent report on amikacin liposome inhalation suspension described an HP-like radiologic pattern [11], its pathologic findings were completely different, showing acute lung injury and pulmonary alveolar proteinosis (PAP) due to macrophage overloading from inhaled liposomes rather than an adaptive immune reaction. Therefore, the mechanism reported in that study represents a non-immune, deposition-related toxic injury, fundamentally distinct from the immune-mediated hypersensitivity reaction observed in our patient. The mechanistic relevance of the liposomal formulation per se thus appears limited in our case, in which PLD was administered intravenously and no PAP-like pathology was identified. In contrast, our case shares clinicopathologic features with epirubicin/cyclophosphamide-associated HP-type DI-ILD [12], in which lung injury was attributed to a combination of anthracycline-induced oxidative epithelial stress and T-cell-mediated hypersensitivity through the pharmacologic-interaction concept. These observations suggest that the anthracycline moiety, rather than the liposomal carrier, is the most likely immunogenic trigger.

Therapeutic response also supports an immune-mediated process. Rapid clinical and radiologic improvement occurred after corticosteroid initiation, consistent with prior reports that HP-like DI-ILD generally shows a favorable outcome following discontinuation of the offending drug and immunosuppressive therapy [5,6,12,13]. Although a surgical lung biopsy was not performed due to the patient’s condition, the combination of compatible radiologic, cytologic, and histopathologic findings provides robust evidence for the diagnosis.

This case underscores two important clinical implications. First, even agents regarded as relatively safe, such as PLD, can induce severe immune-mediated pulmonary toxicity. Second, early recognition of HP-like patterns and timely initiation of corticosteroids can lead to complete remission. Clinicians should therefore remain alert to new pulmonary infiltrates arising during PLD therapy, particularly when accompanied by cutaneous eruptions or systemic symptoms, as these may represent a harbinger of HP-like DI-ILD. Early recognition of initial respiratory symptoms and prompt discontinuation of the offending drug are essential precautions to prevent progression to severe pulmonary toxicity.

## Conclusions

PLD can cause HP-like drug-induced interstitial lung disease. Prompt recognition and appropriate corticosteroid therapy may achieve favorable outcomes. Clinicians should remain vigilant for this rare pulmonary toxicity.
